# Exploring companion robots for children with autism spectrum disorder: a reflexive thematic analysis in specialist dental care

**DOI:** 10.3389/frobt.2025.1659784

**Published:** 2025-12-12

**Authors:** Sofia Thunberg, Erik Lagerstedt, Anna Jönsson, Anna Lena Sundell

**Affiliations:** 1 Interaction Design and Software Engineering, Department of Computer Science and Engineering, Chalmers University of Technology and University of Gothenburg, Gothenburg, Sweden; 2 Linguistics and Theory of Science, Department of Philosophy, Linguistics and Theory of Science, Gothenburg University, Gothenburg, Sweden; 3 Department of Pediatric Dentistry, The Institute for Postgraduate Dental Education, Region Jönköping County, Jönköping, Sweden; 4 Centre for Oral Health, School of Health and Welfare, Jönköping University, Jönköping, Sweden

**Keywords:** companion robots, pedodontics, dental care, children, autism spectrum disorder, human–robot interaction

## Abstract

**Introduction:**

As robotic technologies become increasingly integrated into care settings, it is critical to assess their impact within the complexity of real-world contexts. This exploratory study examines the introduction of a robot cat for children with Autism Spectrum Disorder (ASD) in a specialist dental care unit. Children with ASD often face challenges in dental care, including anxiety, sensory sensitivities, and difficulty with collaboration. The study investigates if a robot cat can provide psychosocial support to the patients.

**Methods:**

Ten patients, aged 5–10, participated in the 12-months study, each undergoing one baseline session without the robot and 3–5 subsequent visits with the robot, yielding 37 sessions of video data.

**Results:**

Reflexive thematic analysis revealed three key themes: the robot cat can *enhance training and treatment*, robot cats can serve as a *beneficial but a non-essential tool*, and robot cats can sometimes *hinder progress in training and treatment*. These findings highlight significant individual variation in how the robot was experienced, shaped by context, timing, and emotional state. The robot’s role was not universally positive or passive; its effectiveness depended on how it was integrated into personalised care strategies by the dental hygienist, guardians, and the patients themselves.

**Discussion:**

This study underscores the importance of tailoring technological interventions in care, advocating for cautious, context-sensitive use rather than one-size-fits-all solutions. Future work should further explore adaptive, individualised deployment.

## 1 Introduction

Children with autism spectrum disorder (ASD) are at a higher risk of developing cavities, periodontal disease, and dental trauma than those without ASD (Meharwade et al., 2021). They may also struggle with maintaining a dental care routine at home, such as brushing their teeth (Pilebro and Bäckman, 2005; Octavia et al., 2023), and often face challenges when visiting a dental hygienist, including difficulties with collaboration and concentration (Marshall et al., 2007; Stein et al., 2012). These factors can contribute to increased stress and anxiety related to dental care (Park et al., 2022; Tang et al., 2023). In Sweden, all children have access to free public dental care tailored to their individual needs. However, children who experience difficulties collaborating in public dental care are referred to a specialist dental care (SDC) unit. This unit helps them gradually adapt to dental visits through personalized treatment plans. Various supportive techniques are common, including stress reduction tools (e.g., stress balls) (Linthoingambi et al., 2022), sensory aids for comfort (e.g., sunglasses or weighted blankets) (Cermak et al., 2015), the “tell–show–do” method (Farhat-McHayleh et al., 2009; Elicherla et al., 2024), and visual pedagogy (e.g., image-based support) (Bäckman and Pilebro, 1999; Pilebro and Bäckman, 2005).

Behavioral and psychosocial interventions are the primary approach for supporting children with ASD, focusing on fostering development and adaptation by teaching essential social and communication skills. Companion robots, such as robot cats, have been designed to provide psychosocial support, and previous research has shown promising results in their use for children with ASD, such as by increasing concentration (Kwon et al., 2015). Furthermore, there is a call in the dental community for novel approaches to help children with ASD (Stein et al., 2014; Duker et al., 2019; Park et al., 2022). Therefore, we wanted to investigate if these robots could serve as a tool in a dental setting.

In this exploratory study, we examined whether a robot cat, using the Joy for All platform, could provide psychosocial support to children aged 5–10 years with ASD during dental visits over the course of 1 year. Ten participants, all familiar with the dental hygienist, were recruited for the study. Data collection included video recordings of one baseline session without the robot, followed by 3–5 visits over the next year, resulting in a total of 37 sessions and 11.55 h of video data. As robots are increasingly proposed to serve a variety of functions in various care domains, it becomes particularly important to investigate what the impact of introducing such technology can be in such contexts. Given the “messy” nature of many real-care situations, it is not enough to rely on the general phenomena observed in different contexts; instead, it is necessary to take the rich variety and complexity of the situations where the robots are to be deployed into serious consideration. This raises high baseline demands of robustness to handle diversity and a continuous demand for monitoring that no harm is introduced by the disruption. Hence, the video material was analysed using reflexive thematic analysis (TA), an approach that emphasizes research workers’ self-awareness and critical reflection throughout the analytical process, to bring the complexity of the interactions to the limelight.

## 2 Related work

### 2.1 Children with autism spectrum disorder in dental care

People with ASD (Association, 2013) generally experience poorer oral and dental health than individuals without ASD, which is partly due to challenges with cooperation and a higher need for general anesthesia during treatment (Corridore et al., 2020; Ferrazzano et al., 2020). In Sweden, dental care is a universal right provided on equal terms under the Dental Care Act (1985:125)^1^, and all children are entitled to necessary dental care free of charge until 19 years of age (Social Services Act, 2001:453^2^). When public dental care providers are unable to meet a child’s specific needs, they may refer the patient to an SDC unit for children. At home, limited verbal communication and mutual understanding between the child with ASD and their guardians can hinder daily oral hygiene practices such as brushing and flossing (Stein et al., 2012; Marion et al., 2016). Difficulties in communication may also prevent children from effectively expressing pain, whereas their sensory perception of pain and touch may be atypical. In the dental setting, communication barriers between the patient and dental professionals can pose safety risks—especially when the patient experiences pain or fear but cannot express it, potentially resulting in behavioral outbursts. Additionally, social challenges may interfere with the development of a trusting relationship with dental staff. Patients with ASD often avoid eye contact and may not respond to verbal explanations of procedures. They may also feel discomfort due to the physical proximity required during dental treatment. Marshall et al. explored factors influencing cooperation among children with ASD during dental visits. The findings showed that 65% of children were uncooperative during regular visits, whereas during emergency visits, the children were 100% uncooperative, highlighting the importance of predictability for the patients (Marshall et al., 2007).

Treating patients with ASD can be challenging for dental professionals due to a range of sensory and behavioral difficulties. Many children with ASD struggle with changes in routine, which can make both initial visits and follow-up appointments stressful, especially when new treatments are introduced. Even minor changes in the dental environment can feel overwhelming. Patients may also be sensitive to variations in sound, lighting, smells, tastes, and physical contact with instruments or dental staff (Balian et al., 2021). Due to these challenges, dental professionals often use specialized behavior management strategies, including visual communication, reward systems, and behavior shaping techniques (Bäckman and Pilebro, 1999; Zerman et al., 2022), which have been shown to improve both compliance during dental treatment and oral hygiene practices at home (Pastore et al., 2023). Various supportive techniques are also used, including stress reduction tools (Linthoingambi et al., 2022), sensory aids for comfort (Cermak et al., 2015), and the “tell–show–do” method (Farhat-McHayleh et al., 2009; Elicherla et al., 2024). Research on how to improve the dental situation for children with ASD has grown in recent years, and there is a call in the dental community for novel approaches to help children with ASD (e.g., Stein et al., 2014; Duker et al., 2019; Park et al., 2022).

### 2.2 Companion robots for children with autism spectrum disorder

Interaction with robots could be beneficial for children with ASD by fostering communication and social interaction. Studies even suggest that children with ASD could interact more easily with a robot partner than with a human (e.g., Syriopoulou-Delli and Gkiolnta, 2022; Šimleša et al., 2022). A recent paper (Dubois-Sage et al., 2024) aimed to explain the benefits of robots by referring to two theoretical perspectives: the social motivation theory (Chevallier et al., 2012) and the social cognition theory (Baron-Cohen, 1995). According to the social motivation theory, individuals with ASD may prefer interacting with nonhuman agents such as robots rather than humans. This increased motivation to engage socially could enable them to accumulate positive social experiences, potentially reducing their social challenges over time. Meanwhile, the social cognition theory emphasizes that people with ASD often participate less in social interactions due to difficulties in comprehending complex social cues. A robot, being simpler and more predictable, could alleviate these challenges, thus encouraging greater social engagement. In this sense, robots serve as appealing, simplified, and reliable social partners, fostering enhanced interaction among individuals with ASD. That being said, there are large variations in the abilities and needs among individuals with ASD, while neurotypical individuals could also benefit from interacting with a robot. These are both heterogeneous groups with individual needs that potentially, in some contexts, could enhance social interaction through and with a robot (Hundt et al., 2024).

Robots come in many different forms, and one common strategy is to mimic a familiar morphology, where household pets, such as cats, are often used. Due to logistical, allergy-related, or safety constraints, robot cats have emerged as viable alternatives in care settings where live cats are not feasible. Although live animals remain preferable when available (Steen et al., 2025), robotic pets have demonstrated comparable effectiveness to traditional animal-assisted therapies (Kramer et al., 2009), offering substantial social–emotional benefits. Robot-assisted therapy for children with ASD, particularly using touch-based interaction (Burns et al., 2021), has advanced significantly in the recent decades (Libin and Cohen-Mansfield, 2004; Billing et al., 2020). Children with ASD can learn social behaviors from these interactions (Raptopoulou et al., 2021) and may even be more motivated to participate in the intervention as a result of the presence of the robot (Rabbitt et al., 2015; Pennisi et al., 2016). The robots can further improve emotional recognition and expression (Pokorny et al., 2021), collaboration (Ali et al., 2020), and social interaction (van Otterdijk et al., 2020; Alabdulkareem et al., 2022), and interventions with companion robots have been recommended to be carried out on a much larger scale than they currently are (Huijnen et al., 2017).

One study involving a robot cat found increased concentration among children with ASD, driven by greater interest and attention than that in traditional therapies (Kwon et al., 2015). Some children even engaged with the robot immediately, which is unusual. Direct, affectionate interactions—such as hugging or facial rubbing—were common, whereas severe autism symptoms initially correlated with aggressive behaviors toward the robot. However, as treatment progressed, the intensity and frequency of the negative behavior appeared to decrease. Another study evaluated a social robotic platform based on soft actuation to promote physical interaction for children with ASD (Pinto-Bernal et al., 2022). The results showed no significant influence on the patient’s performance in the activity; however, the clinicians remarked that encouragement and motivation increased when the children were allowed to interact with the robot physically.

## 3 Methods

This exploratory, interdisciplinary study combined pediatric dentistry with human–robot interaction (HRI). The study started with a standard dental hygiene visit, which served as a baseline. For subsequent visits, the patient and guardian were provided with updated visual support materials that included the introduction of the robot cat, which was used as a *static exploration* familiarization method to prepare the patient for stimuli (Wallbridge et al., 2024). At each session, the patient and guardian entered the treatment room where the robot cat was positioned in the dental chair. The dental hygienist then offered the patient the option to hold the cat in their lap during the appointment; for example, the dental hygienist encouraged the patient to pet the robot cat as a kind of *capability demonstration* (Wallbridge et al., 2024). If the patient declined or changed their mind during the session, the robot cat was placed either on a nearby table or in the guardian’s lap. The dental procedures followed the routines established in the patient’s previous training or treatment sessions. Each session concluded with the patient receiving a reward and rating the visit using the autism feeling chart. The sessions involving the robot cat were repeated over the course of 3–5 visits per patient across 1 year, resulting in a total of 37 sessions. Data collection took place between October 2022 and August 2024.

### 3.1 Material

**FIGURE 1:**
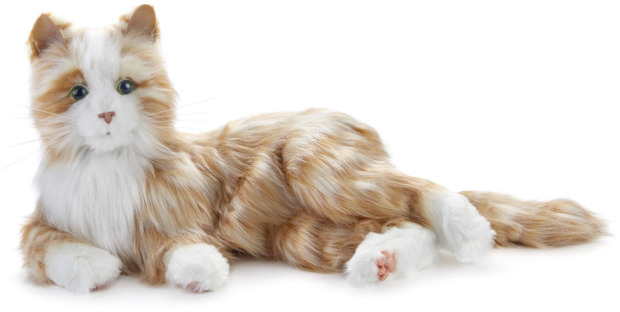
The Joy for All robot cat. ©Ageless Innovation.

The robot platform used was the *Joy for All* cat by Ageless Innovation (see Figure 1). This is a simple robot model equipped with movement, touch, and sound sensors. In response to the sensor input, the cat can purr, meow, lick its paw, and roll onto its back. When inactive, it simulates sleep by closing its eyes.

### 3.2 Video recording

**FIGURE 2:**
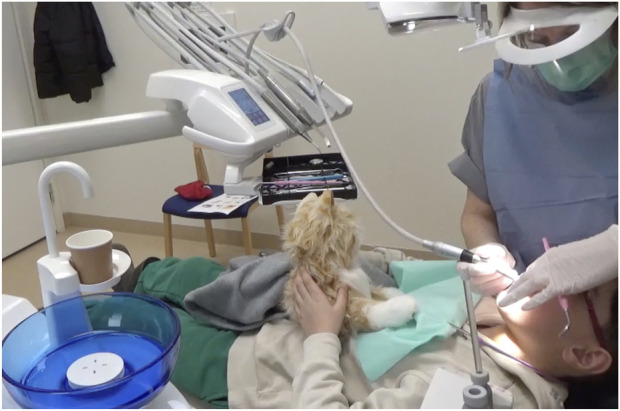
Example from the video showing ongoing treatment with patient P7 and the robot cat.

Video recordings were captured using a camera positioned beside the dental chair, covering the central areas of the treatment room (see Figure 2). The dental hygienist initiated the recording before bringing the participant from the waiting room and stopped it once the procedure was completed. In total, 11.55 h of video footage were collected.

### 3.3 The autism feeling chart

**FIGURE 3:**
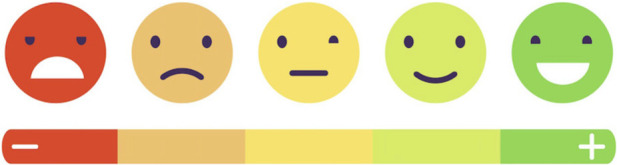
Autism feeling chart; from the left to the right, from red = very bad to green = very good.

The autism feeling chart (see Figure 3) is commonly used with children who are nonverbal or have limited communication abilities. Following each session, the dental hygienist invited the patients to evaluate their experience by pointing to a rating on the chart.

### 3.4 Participants

The 10 patients (all boys, no withdrawals) are presented in Table 1. Several of the participants have multiple diagnoses, such as attention deficit hyperactivity disorder (ADHD), psychomotor development retardation (PDR), tuberous sclerosis complex (TSC), developmental language disorder (DLD), and intellectual disability (ID). Some of the participants have undergone early intensive behavioral treatment (EIBI) (e.g., Healy and Lydo, 2013), which is an evidence-based therapy that has been shown to offer effective support in addressing the problematic symptoms of children and youth with ASD. The approach typically includes *discrete trial training*, *pivotal response training*, and *intensive behavioral intervention*. Common elements of all forms of effective EIBI are high treatment intensity, involvement of family and teachers, and delivery by properly trained therapists. However, in Sweden, dental care is not an active partner in this training.

**TABLE 1:** Participant data.

ID	Age	Sessions	Robot exp.	Diagnosis	Medications	EIBI	Pre visits	First visit
P1	8	3	Dislike	ASD (2) ADHD (n/a) DLD	Melatonin Methylphenidate Promethazine		13 (10)	Jun 2018
P2	6	4	As toys	ASD (n/a) PDR Sleep disorder	Melatonin Atomoxetine		3 (3)	May 2019
P3	9	3	Dislike	ASD (2–3) ADHD (2) ID (1–2)	Dexamfetamine		11 (5)	Dec 2019
P4	10	4	None	ASD (n/a) ADHD (n/a) TSC Epilepsy	Oxcarbazepine	Yes	21 (8)	Mar 2015
P5	5	3	None	ASD (2–3)	Melatonin Methylphenidate Alimemazine	Yes	5 (1)	Jan 2022
P6	5	5	None	ASD (n/a)	Alimemazine Risperidone	Yes	4 (1)	Dec 2021
P7	9	4	As toys	ASD (2–3) ID (n/a)	None		11 (1)	Mar 2020
P8	6	4	None	ASD (2) ID (n/a)	None		6 (2)	Mar 2021
P9	8	4	Likes	ASD (n/a) ADHD (n/a) ID (1)	Dexamfetamine		13 (5)	Mar 2020
P10	7	3	Toys	ASD (2–3)	None		7 (2)	Feb 2021

ID = participant number; age = age at the beginning of the study; sessions = times with the robot cat in the study; robot exp. = previous experience with robots; diagnosis: level 1 = mild, level 2 = moderate, level 3 = severe, and n/a = ungraded; medications = active substances; EIBI = early intensive behavioral intervention; pre visits = the first number is visits to the dental hygienist, and the number within parentheses is visits to the dentist at the specialist dental unit before the study; first visit = first visit at the specialist dental unit.

#### 3.4.1 Medications

Most patients who participated in this study were regularly using various kinds of medication (see Table 1 for a list of active substances). The purpose of these medications is to treat symptoms such as sleep disorder, nausea, anxiety, hyperactivity, schizophrenia, and epilepsy, and to increase impulse control, attention, and concentration. These kinds of symptoms are common among people with diagnoses such as ASD and ADHD. It is, however, important to emphasize that these medications are *not* intended (nor able) to “treat ASD” itself or similar disorders.

### 3.5 Ethical considerations

This study was approved by the Swedish Ethical Review Authority (reference nr. 2022-02933-01), and several ethical measures were implemented. Participants were recruited by their regular dental hygienist and provided with information to take home—addressed to both guardians and patients. The patient information was age-appropriate and included visual supports to aid understanding. Written informed consent was obtained from the guardians for participation in the study. A separate written consent was also collected regarding video recordings and their potential use beyond the research team (e.g., inclusion in publications or conference presentations). On the first day of participation, oral assent was obtained from each patient. All participants remain anonymous and are identified by pseudonyms. Collected data have been securely stored using a two-factor authentication system, in accordance with university data protection policies.

### 3.6 Analysis

The video material was analysed using reflexive TA. Reflexive TA, developed by Braun and Clarke, is a qualitative method that emphasizes the research worker’s active role in identifying and constructing themes from data (Braun and Clarke, 2019). Unlike structured approaches that rely on codebooks or aim for coding reliability through multiple coders, reflexive TA treats research worker subjectivity as a valuable resource rather than a threat. It encourages a fluid, recursive process of coding, theme development, and interpretation that aligns with a qualitative research paradigm. Central to this method is reflexivity: the research worker must continuously reflect on their assumptions, decisions, and theoretical orientations throughout the analysis (Braun and Clarke, 2021).

Themes in reflexive TA are not seen as simply emerging from the data; instead, they are generated through deep engagement, interpretation, and meaning-making. These themes represent patterns of shared meaning, underpinned by a central organizing concept, and are constructed at the intersection of the data, research workers’ perspectives, and analytic process. Reflexive TA is adaptable, supporting both inductive and deductive approaches, but it requires transparency and theoretical coherence. Braun and Clarke advocate moving beyond rigid proceduralism, urging research workers to embrace the creativity, flexibility, and conceptual richness of qualitative analysis while being thoughtful and deliberate in their methodological choices. In recent years, reflexive TA has been increasingly used in qualitative health-research domains, such as for investigating referral behaviors in Nigerian postnatal care (Campbell et al., 2021), for exploring mental health in educational settings (Byrne, 2022), and for palliative care (Braun and Clarke, 2024b).

Limitations of reflexivity include noting that it is influenced by personal biographies, academic training, institutional contexts, and emotional dynamics (Mauthner and Doucet, 2003). Mauthner and Doucet advocate for greater transparency regarding the assumptions embedded in research methods and for creating dedicated spaces and structures to support reflexive practice. We engage with this through our positionality in the following section and by using the Reflexive Thematic Analysis Reporting Guidelines (RTARG) (Braun and Clarke, 2024b; Braun and Clarke, 2024a). To analyze the video data, we engaged in several forms of reflexivity, primarily: i) *introspective reflexivity*, which involved drawing on our own self-understanding to inform interpretations and connect knowledge with both the participant and research worker experiences within their social context; ii) *intersubjective reflection*, focusing on the relationship between the research worker and participant; and iii) *mutual collaboration*, recognizing participants as co-research workers who actively contribute to the co-construction of knowledge (Finlay, 2002).

The reflexive TA process started when the first, third, and fourth authors held regular meetings during the year of data collection to discuss the participants’ progress and usage of the robot cat, reflecting on the video material that the first author viewed after each session and how the third author had experienced the sessions. The larger project (Thunberg et al., 2025^3^) further involved interviews with guardians (conducted by the first author) that were also integrated into these reflections. During the guardian interviews, the first author asked specific questions related to what she had seen in the videos and encouraged them to reflect on that, integrating the guardians as experts on their children and as co-research workers. When the data collection was finalised, the first two authors watched the video material together while taking individual notes. Each participant, from the baseline video to the last visit with the robot cat, was watched consecutively to follow the process over the year. The authors reflected after each video, after each participant’s 4–6 videos (baseline included), and after certain sequences of importance. The results of the autism feelings chart were integrated into the video analysis, and the first author offered perspectives from the guardian interviews and from previous reflection meetings with the third and fourth authors. Even if this data collection is not specifically included nor reported on in this paper, the insights were unavoidable and strengthened the reflexive TA process to interpret the video data. After this process, the two authors coded their notes individually and reflected on these codes together, one participant at the time. As a next step, the authors combined the codes to themes in a visual code mapping session. A separate reflection on communication modalities and practice of how the robot cat was used by the participants, guardians, and the dental hygienist was conducted at the same session. The themes were then reflected upon together with the third and fourth authors to deepen the dental professional perspective on the HRI findings, especially through the third author’s own experience while collecting the data.

### 3.7 Positionality

Guided by the principles of situated knowledges (Haraway, 1988), feminist human–computer interaction (HCI) (Bardzell, 2010), and recent calls for feminist HRI (Winkle et al., 2023), we actively worked to include diverse perspectives that might otherwise be marginalized. All authors are white, and three members of our research team identify as women. All team members are fluent in the local language and were born in the country where the study is conducted.

The first two authors have a background in cognitive science and HRI, whereas the third is a dental hygienist and the fourth is a specialist dentist in pedodontics. The third and fourth authors have 17 and 18 years of clinical experience, respectively, working with children with ASD. The first, third, and fourth authors planned and conducted the study, whereas the second author offered an extra layer on the analysis as he would not be colored by the study process. The third author collected the video material while conducting the dental training and treatment. None of the authors have their own lived experiences of ASD. As shown in the related work section, there is a substantial body of research on robots for children with ASD in HRI. Yet, we are aware that the field has received critique for not prioritizing the needs of the intended user group, for burdening the caregivers or the robot wranglers (Wright, 2023; Hundt, 2024), and for sometimes worsening the situation for the participants (Hundt et al., 2024). Some studies have contributed to stereotypical use cases, and there is a growing body of research trying to demonstrate that people with ASD have the theory of mind (Gernsbacher and Yergeau, 2019), are empathetic (Kimber et al., 2024), and are willing to reciprocate (Gernsbacher, 2006), in response to previous perceptions. We have continuously reflected on this while planning and conducting the study, made it easy for the participants to put away the robot cat when they want to, and through the analysis process, observed individual effects instead of treating the participants as a homogenous group.

This paper takes on a post-humanist lens on care (Puig de la Bellacasa, 2011; DeFalco, 2020). Care carries a dual meaning, referring both to a mental orientation of concern and to the tangible practices undertaken in response to such concern (Tronto, 1998). Traditionally, “good care” has been understood as inherently human, grounded in a view of individuals as relational, interdependent, and morally responsive, rather than as autonomous and self-sufficient actors (Held, 2018). In contrast, post-human perspectives on care challenge the human-centered foundation of this view. From this standpoint, “good care” is not necessarily human care (DeFalco, 2020) but rather emerges from a recognition of human beings as provisional, contingent, and fundamentally entangled with both animal and technological forms of existence. Humans, in this view, are considered one companion species among many, reliant on more-than-human forms of care for survival and identity. Puig de la Bellacasa extends this notion by arguing that care is not solely a human activity (Puig de la Bellacasa, 2011). She describes nonhuman agents and ecologies as participating in a “living web of care,” emphasizing how care circulates through the natural world.

## 4 Results

In this section, we begin by describing the current practice, followed by the presentation of the three effect themes: robot cats can *enhance training and treatment*, robot cats can serve as a *beneficial but non-essential tool*, and robot cats can sometimes *hinder progress in training*. Finally, we present different communication modalities that the patients, guardians, and the dental hygienist used during interaction with the robot. Participants will be referred to as P1–P10, and quotes in Swedish are freely translated to English.

### 4.1 Current practice

After referral from public dental care, the patients are called for a first visit to the SDC unit, where they receive image support showing the steps that will be taken during their next visit. In this image support, simple elements, such as a treatment chair, lamp and mirror, and toothbrush, are shown in the order that the patient will experience them to aid their planning of the visit. At the first visit, the dental professionals ask the guardians general questions about health and oral and dental health and specific questions about how the patient communicates; if the patient uses visual support and signs; if the patient is sensitive to light, sound, smell, taste, or touch; if the patient has any special interests; and how the patient is best rewarded. Rewards can be a small toy in the “reward box,” a round of applause, or watching a specific YouTube clip. An individual treatment plan is thereafter created together with the guardian based on the patient’s treatment needs and conditions. The guardian and patient are informed that the patient will always meet the same staff during the visits and that regular visits may be required over a long period of time to achieve the set goals. For subsequent visits, the guardian prepares the patient at home with the help of the image support provided during previous visits. The patient and guardian are brought in from the waiting room to the treatment room punctually to reduce the risk of the patient becoming upset or losing concentration. The treatment room, if possible the same for all visits, is sparsely furnished, and the door is kept closed during the visits. With the help of the image support and the “tell–show–do” method, dental professionals tell what should happen next in simple words in short sentences and use support signs if necessary. Various aids can be offered to those who wish for them, such as items that provide security or distraction. Examples include stress balls, pop-its, tangles, weighted blankets, singing, music or films on the guardian’s phone/iPad, and stuffed animals. Then, the training or treatment begins, according to previous agreements. The patient receives the reward when the visit ends. In this study, the robot cat was always offered and sometimes in combination with other tools that the patients had with them (e.g., earphones or fidget spinners). The dental hygienist (third author) used primarily the “tell–show–do” method, image support, singing, and counting down during the sessions. As rewards, the patients were sometimes applauded by the dental hygienist and the guardian, and were always offered to take a small toy from the reward box.

The goals of the SDC practice can be, first, to help the child become comfortable being in the dental room. It is common that the patients are stressed about the visit and the new routines that it requires and, therefore, are uncomfortable entering or staying in the dental room. When this is achieved, the practice moves on to the next phase, which is to undergo dental training. This includes multiple steps, such as sitting in the treatment chair, brushing teeth, and allowing the dental hygienist to use different dental tools inside the mouth. The training can take several years, with 3–5 visits a year, before the patient is comfortable enough to undergo treatment. Treatment is the final goal, where the patient collaborates with the dental hygienist and is comfortable to undergo the treatment steps that are necessary (e.g., removing dental calculus). In addition, this part can take a longer time for the patients to get comfortable with, and the patient may go backward in progress when new treatments are introduced. The long-term goal of the SDC unit is not for the patients to return to public dental care nor for the dental sessions to be more effective. The sessions are always scheduled to give enough time for the patients to become comfortable and to be able to address issues that arise (approximately 50 min). The patients almost always remain in the SDC unit and often continue to do so throughout adult life. Therefore, the goal of introducing the robot cat is not to make dental care more time-efficient but to aid the patients with being in the situation and undergo training and treatment.

In SDC, there are typically a lot of things the care professionals must keep in mind simultaneously (adjusting care to the individual patient’s needs), while presenting a calm, collected, professional, empathic, and assuring facade. The care professionals are already relying heavily on reflective practices, continuously evaluating their own behaviors and decisions for each of the patients. The care professionals typically have to be pragmatic and flexible in adapting to situations as they pan out. Situated problem-solving (potentially partly collaboratively with patients or guardians) is typical for this care work, where “tools” and “toolbox” are useful metaphors for the dental professionals. They are often used by explicitly explaining that “I do not have any more tools in my toolbox,” which can in itself be a useful strategy in some situations when complex issues arise that the professional do not know how to solve during the session. There might also be sudden unexpected opportunities to adjust to. For example, P5 brought his own tooth brush for session 3 (second session with the robot cat), at which point the dental hygienist removed the tooth brush provided by the care facility from the prepared tools. It is a small adaptation but still an example of how the situation can dictate the practice rather than the other way around.

### 4.2 Effects of the companion robot

**FIGURE 4:**
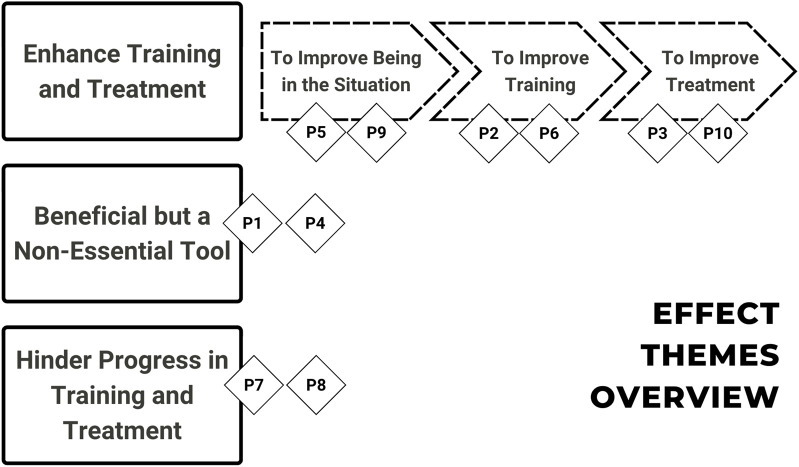
Overview of the effect themes *enhance training and treatment*, *beneficial but a non-essential tool*, and *hinder progress in training and treatment* with patients P1–P10.

To better understand the effects of introducing the robot cat in SDC, we will introduce each case to provide context for our observations. The cases are organized in three broad themes (see Figure 4 for an overview): the cases where the robot cat *enhances training and treatment*, the cases where it was *beneficial but a non-essential tool*, and the cases where the robot cat might *hinder progress in training and treatment*.

#### 4.2.1 Enhancing training and treatment

There were signs that the situation for six of the ten patients in this study (P2, P3, P5, P6, P9, and P10) improved at least partly due to the robot cat. Three of these patients (P2, P5, and P6) are also among the youngest of the participants in the study (5–6 years old), and the three of them are all at the stage of getting used to being in a dental situation (early training).

##### 4.2.1.1 To improve being in the situation

**FIGURE 5:**
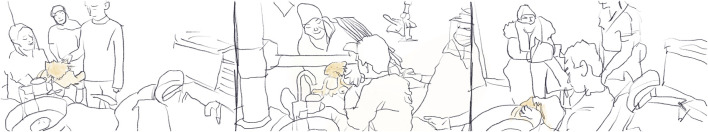
Three frames with P9, from the left to the right: from sessions 1, 2, and 4. 1) P9 meets the robot cat for the first time, cautiously poking the robot; 2) P9 has had the robot cat in his lap but gives the robot to his guardian; and 3) P9 has been asked about keeping the robot cat for the following visits, and he himself brings the robot to his lap to cradle and pet it.

In the baseline session without the robot cat, P9 (see Figure 5 for sketched frames) is hesitant about sitting in the dental chair and is worried when it moves. When the robot cat is introduced, he initially shows a slight interest in the cat’s name and pets it but does not want to hold the cat during the visit. For the following visits, he does not show much interest in the cat even though both the dental hygienist and the guardian try to demonstrate how the robot can be petted. At the fourth session with the robot cat (which is the final session in the study for this patient), the patient shows no interest in the robot cat until the dental hygienist informs him that this is the last time for the study and wonders if he would like to continue with the cat. The patient then jumps out of the dental chair and approaches the robot cat lying on a chair to pet it, saying he likes it. Both the guardian and the dental hygienist become a bit surprised by this. This case indicates that it might not always be evident from an outside observer when the patient gains from the robot cat interaction. The patient might not need to directly interact with the robot but prefer to have it in the environment as it has been introduced as a part of it.

Another patient who appears to benefit from the cat in terms of support for being in the dental situation is P5. P5 and P6 are twins and are facing similar difficulties; they are both undergoing EIBI, and they are both relatively new to the SDC unit (one previous visit to the dentist and 4–5 visits to the dental hygienist).

Throughout the baseline session (without the robot cat), P5 wore his outdoor clothes. He quickly engaged with a simple wooden puzzle themed around farm animals, and the guardian joined in this activity while the dental hygienist prepared the equipment to use in the session. P5 kept his focus on the puzzle and imitated the sounds of the animals together with, and encouraged by, the adults in the room. When he appeared to be satisfied with this play and was looking around the room, the dental hygienist (together with the guardian) proposed that he should sit down in the dental chair. The patient’s response to this was to turn back to the puzzle, starting to manipulate its pieces. When the guardian indicated that the time for play was over, the patient pushed the guardian away, persisting in playing with the puzzle a few moments more before turning away and starting to explore the rest of the room (carefully avoiding the dental chair). To get the patient to focus on the dental care situation, the guardian goes through the plan with the steps for the session (part of the “tell–show–do” method) with him. He pays attention to this but starts to cry as it becomes evident that there is no escape from the dental situation. The guardian sits down in the dental chair, grabs the patient, and holds him in place. The interaction is quite complex at this stage. The patient is, on the one hand, struggling, crying, and resisting (e.g., trying to walk away and squirming), but he is, on the other hand, also cooperating by opening his mouth when instructed to do so (and he also appears to hold back on his physical resistance). After a few minutes of dental treatment training, the patient is released and collects himself quickly as he wanders the treatment room.

As P5 enters the SDC room for the next session, the robot cat lies in the dental chair. P5 walks up to the chair and looks at the robot cat for a short while but does not show any sign of deeper interest. Before sitting down for training and treatment, the guardian and the dental hygienist encourage P5 to pet and interact with the robot cat in other ways. This interaction continues for just over half a minute, at which point P5 starts to lose interest again, and, much like in the previous session, the guardian sits down in the chair holding the patient in her lap. P5 is offered to hold the robot cat but pushes it away, and the training and treatment follows a similar pattern as that of the previous session. After P5 is released again, he shows no interest in the robot cat for the rest of the session despite the adults’ encouragement for interaction one more time.

For the third session, P5 acknowledges the robot cat but is initially not particularly interested in it. However, when it was getting time for the training and treatment, he does start to interact with it as he is getting pressured to approach the dental chair. The training and treatment is similar as that in previous sessions, but P5 is released fairly quickly. The adults try to persuade P5 to return to the chair to continue, and at this (apparently stressful) point, he notices the meowing of the robot cat and starts to interact with it. This continues for approximately half a minute, and then, P5 is physically brought back to the chair to complete the training and treatment. After the completion, P5 is released and calms down immediately, and he shows no interest in the robot cat for the rest of the session.

For the final session, P5 acknowledges the robot cat upon entering the room but largely ignores it for the rest of the session. When it is time to start the training and treatment, the robot cat is moved to a less prominent place and turned off to not move or make sounds. The situation appears to be similarly stressful for P5 compared to previous sessions, but he is more able to contain his stress this time, and the adults primarily rely in the printed plan for the session (part of the “tell–show–do” method) to communicate with P5 what is happening. Toward the end of the session, the dental hygienist brought back the robot cat and encouraged P5 to interact with it, but P5 showed almost no interest in doing so at this point. For P5, the robot cat does not seem to be particularly interesting in itself, and it is not clear that it is improving the situation in general. However, P5 still appears to benefit from its presence as it provided opportunities for the patient to use it as a tool to create moments of respite when preparing or recovering from stressful moments. Although the situation of P6 is similar to that of P5, it was possible for P6 to progress a little further throughout the year and start training on dental training to a larger extent.

##### 4.2.1.2 To improve training

**FIGURE 6:**
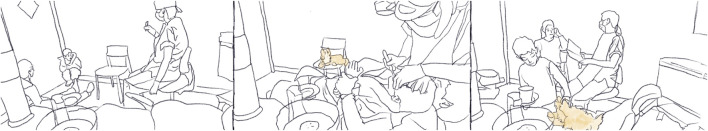
Three frames with P6, from the left to the right: from baseline to sessions 2 and 3. 1) P6 does not want to participate in the training and sits on the floor; 2) P6 is being held by his guardian in the chair while the dental hygienist is brushing his teeth; and 3) P6 is petting the cat while the adults talk.

The focus for P6 (see Figure 6 for sketched frames) in the earlier sessions is on training to be in the room or situation at all, but toward the end of the year, progress was made to the point where the training for getting dental treatment could start. In the earlier sessions, the patient is explicitly and clearly unwilling to enter the room at all; at times, the guardian needs to carry the patient for him to enter. During the treatment, the patient needs to be held in place in the dental chair by the guardian. In the later sessions, the patient enters the room by himself and spontaneously sits for a short time in the dental chair. However, the guardian still has to sit in the chair with the patient while training for the dental care.

The patient’s initial reactions to the robot cat was to ignore it until it started moving, at which point P6 stared at the robot and then went over to it to investigate. Both the guardian and the dental hygienist were actively encouraging this interaction. After a few sessions, the robot cat is used as a way to encourage the patient to enter the room by himself. The patient also starts to occasionally use the robot cat as a focus for his attention when being in a stressful situation, potentially as an acceptable escape. In addition, the patient starts to show some interest in the robot cat after each session when the dental hygienist and the guardian are having a post-session conversation. Apart from the aforementioned potential factors for explaining this improvement (age and EIBI), P6 also started with a new medication (risperidone) during that year.

P2 was also among the youngest (6 years old) patients part of this study, but he was already comfortable being in the room and the dental chair in the baseline session (although he showed some initial unwillingness to approach the dental chair). For that reason, it was possible to focus on the training for getting dental care. The dental hygienist explained all the actions slowly and carefully, and the guardian actively supported the dental hygienist and the patient. The first session with the robot cat was quite similar as the baseline session, as P2 largely ignored the robot cat. After the dental training, before leaving the room, P2 did interact with the robot cat for roughly a minute while encouraged by the guardian. In the following sessions, he showed much more spontaneous interest in the robot cat, and he kept it in his lap during training. During these sessions, he showed less resistance against the dental hygienist and fiddled with the robot cat to keep his hands busy (providing a constructive source for stimming). Although he was not constantly fiddling with the robot cat, he did seem to enjoy keeping it in his lap and having the option to interact with it when he felt like it. The latter sessions were generally much more harmonious.

##### 4.2.1.3 To improve treatment

**FIGURE 7:**
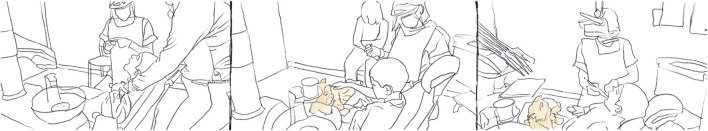
Three frames with P3, from the left to the right: from baseline and sessions 1 and 2. 1) The guardian holds the patient during the whole baseline session; 2) P3 holds the robot cat, and the guardian sits on the side; and 3) P3 has the robot cat in his lap and lies further down in the chair than in previous sessions.

The last two patients (P3 and P10), both of whom appeared to benefit overall from the robot cat, were able to receive dental treatment during the sessions. P3 (see Figure 7 for sketched frames) tends to be nervous around sounds and movements, particularly when they are unexpected. He starts the sessions by inspecting the room and the chair, and when sitting down, he is typically quite active with his hands and moves his head a lot. When the leaning of the dental chair is adjusted, the guardian has to hold on to him, as he worries that he will fall, and he is also concerned about lying down in the chair. Through the whole baseline session, he wants his guardian to hold on to him while he is in the chair. When the robot cat is introduced, he quickly gains an interest in it, and he keeps it in his lap throughout the treatment. Already during the first session with the robot, he does not ask his guardian to hold him, which surprises her. P3 instead focuses on the robot cat. The sounds and the movement of the robot cat do not appear to bother him, and he generally likes to interact with it between the steps of treatment. The robot cat also becomes a focus for his attention throughout the sessions, whereas he initially appeared to pay attention to many different things in the room. For the second session, we see him in Figure 7 lying even further down in the chair (which previously was one of his main worries) with the robot cat in his lap.

Compared to P3, P10 appeared much less anxious, but he was still quite active. In the baseline session, he walked over and sat down in the dental chair without any issue, but while in the chair, he was moved a lot and investigated the chair and the tools. When the robot cat was introduced, he took an interest in it immediately, and he kept it on his lap throughout the treatment sessions. In the first session with the cat, there were times when his attempts to interact with the robot cat interfered somewhat with the treatment. After a while, he was satisfied with holding and petting the cat during treatment, and interacting more with it when the dental hygienist was not actively working in his mouth. Again, the presence of the robot cat appeared to constitute a focal point for the patient to attend and was something for the patient to keep his hands busy with.

##### 4.2.1.4 Common themes in enhancing cases

Although the patient–robot interaction (and how it changed throughout the year) varied to a large extent between patients, there were still some general functions the robot cat provided to various degrees to the different patients who appeared to benefit from its introduction. For many of the patients, the dental situation was quite stressful, and the robot cat could support some of the patients in the stressful situations. For some (e.g., P5), paying attention to the cat became an alternative to participating in the dental care situation, which was still acceptable for the dental hygienist and the guardian. By attending the cat, the patient could get a moment of respite while signaling a need from the adults to back off. These situations were at times intense and complex, but the added complexity of adding the robot cat was, in a sense, in favor of the patient, providing options and freedom for the patient to navigate.

A less dramatic version of this phenomenon was how the robot cat sometimes provided opportunities for the patients to relieve some of their stress. Having an object to focus on to pet and investigate when the situation started to become overwhelming was helpful for some patients (e.g., P6). An even less dramatic version of this was, for instance, seen to a large extent for P10, where the situation was not particularly stressful but there was still a need to address the patient’s restlessness. The patient could keep their hands busy with the robot cat, and having it rest on his lap was also likely providing some comfort. Another function that the robot cat provided for some patients was as a reward. Some patients (e.g., P8) were not particularly interested in the robot cat during the sessions but started to interact with it as soon as the session was over. This phenomenon is a good reminder that the impact of the various tools and methods introduced might not be easily detectable if only the actual treatment situation is investigated. This is also relevant for some of the previously mentioned phenomena, such as increased pride and social status in their network of relationships away from the dental situation (e.g., among friends and family), which might only indirectly affect the dental situation.

In conclusion, we saw how the robot cat was a useful artifact for some of the patients. It is not always clear precisely what about the robot cat provides the benefits (but is likely a complex and dynamically changing set of factors), and there are likely other alternatives that could provide some of the same functions—such as something soft to touch or something to fiddle with—but the robot cat has been shown to be at least one of the potential tools for a dental hygienist to consider.

#### 4.2.2 Beneficial but a non-essential tool

Two of the patients (P1 and P4) were identified as gaining some beneficial effect of interacting with the robot cat, but it did not appear to be an essential part of their treatment. These are the most long-term patients, being familiar with the facility and the dental hygienist since 2015 and 2018, respectively. This indicates that they had gone through the training period and were already comfortable with the treatment. When the robot cat was introduced, both of these patients immediately accepted the robot and had it in their lap during the first session. In the following sessions, they continued to use the robot. We observe that both patients got used to the robot and continuously used it, and more importantly, they started to see it as a natural part of the dental environment. One can speculate that if the robot would be removed in the future, these patients’ treatment would probably not be affected even if they might miss it.

P1 keeps the robot cat in his lap throughout the sessions. When there is an opportunity (e.g., when the dental hygienist is changing tools), he typically interacts with it briefly by watching it and petting it but will immediately go back to the role of the patient when required. Interestingly, halfway through the first session with the robot cat, P1 mentions that he is bothered by the robot cat (likely due to it moving and mewing in P1’s lap during treatment). It is moved by the dental hygienist to the side of the patient, which P1 was satisfied with. This particular example shows how the patient was comfortable enough to both use the robot cat and also able speak up if it was too much. This allows for joint decisions between the patient and dental hygienist regarding what a comfortable environment for the treatment is.

Similarly, P4 is immediately interested in the robot cat and likes to have it in his lap throughout the sessions. He is able, without any problem, to ignore the cat during treatment, but he uses opportunities to pet or talk to the robot cat. P4 talks a fair bit with the dental hygienist, and part of the conversations is about the robot cat. Throughout the sessions, the robot cat appears to have become a natural part of the environment, and P4 names it “Rut.” In the final session of the study, P4 greets the robot cat at he sits down and puts it in his lap, and when the dental hygienist says “now you [singular] will go backwards” as she changes the mode of the dental chair into the lying mode, P4 corrects her with the statement “Rut as well.” To reiterate, for both these patients, the dental training and care generally went smoothly even without the robot cat. However, introducing the robot cat did not appear to cause any problem, and, if anything, it contributed to a good atmosphere and potentially positive associations.

#### 4.2.3 Hindering progress in training and treatment

For two of the patients (P7 and P8), the introduction of the robot cat did not appear to provide any net positive effect. On the contrary, in these two cases, the introduction of the robot cat appeared to have a negative effect. The work for the dental hygienist became more difficult, which, in turn, made it more difficult for her to provide the same high level of dental care as without the cat.

**FIGURE 8:**
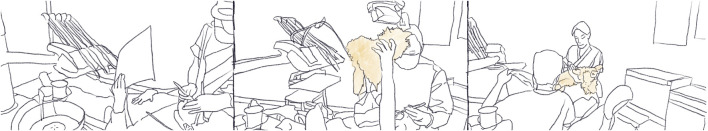
Three frames with P7, from the left to the right: from baseline to sessions 2 and 3. 1) P7 follows the visual image support step by step; 2) P7 wants to look at the robot cat during the whole session and holds it in the air during treatment; and 3) the dental hygienist turns off the robot after too much play with it.

Patient P7 (see Figure 8 for sketched frames) is one of the older patients (9 years old) and has been to the specialist dentist for a longer period but only been to the dental hygienist once before the study started. He actively uses the visual imagery of the treatment and the “tell–show–do” method, following along the sheet describing the order of the procedures as they are carried out. P7 chats a lot with the dental hygienist about the dental care procedures, things in the room, and events happening in his life. The dental hygienist is able to participate in these interactions while still preparing and carrying out procedures (although brief pauses in the procedures happen on occasion, giving the patient a break to refocus). At times, P7 also participates by pressing the buttons that control the dental chair, under the supervision of the dental hygienist. However, P7 is very distractable and tends to quickly switch focus, which makes it necessary for the dental hygienist to very actively engage in the interaction, guiding the attention of P7 in a way that keeps the session sufficiently on track. This allows the patient’s mind and attention to roam, thus preventing exhaustion.

When the robot is introduced, P7 immediately likes the robot cat and is clearly enjoying the interaction. P7 keeps it in his lap, continuously pets it, looks at it, and talks about it. He expresses some occasional worries that the robot cat might get hurt if it is hit with the dental tools but allows the dental care on him. This work is made more difficult for the dental hygienist as P7 keeps trying to look at the robot cat and wants to keep talking about it rather than continuing with the dental procedures. P7 no longer shows any interest in the sheet from the “tell–show–do” method, which was his focal point in the baseline session.

The following sessions proceed much like the first session with the robot cat. P7 has many questions about the robot cat and plays with it during treatment. The focus on the cat makes the patient forget about the treatment (however, not as a distraction that facilitates treatment but as a prevention from starting the treatment session), and the previously used methods are not of interest to him anymore. The dental hygienist becomes frustrated with the situation, and, at one time, she turns off the robot and puts it away. For this patient, the robot cat replaces the sheet from the “tell–show–do” method (that appeared to work quite well for P7), while disrupting the situation in a way that makes the work of the dental hygienist more difficult. Even if the patient enjoys the interaction, it appears to make the treatment sessions worse than before. The robot could probably be removed, potentially allowing the patient to play with it after the session (however, keeping it in the room during the treatment would likely also be quite distracting for P7).

In the case of P8, he is one of the younger patients (6 years old) who were recently remitted to the SDC unit. He has only been there two times before the start of the study, and in the baseline video without the cat, the focus is on training to sit in the chair and have his teeth brushed. Much like P7, P8 sits in the chair without any problem, is quite distractable, and likes to play with the buttons adjusting the chair. In this case, the dental hygienist is also supervising this behavior, showing the patient how to use the buttons. In contrast to P7, P8 does not speak much nor does he show much interest in the sheet from the “tell–show–do” method, but he responds well to the dental hygienist singing a jingle-like song (“brush brush brush your teeth”) while working.

During the session when the robot is introduced, the patient finds it somewhat interesting, petting and interacting with it, and keeps it in his lap for a while. After approximately half the session, P8 shows that he no longer wants the cat in his lap, by gently shoving it away. The dental hygienist picks up the robot cat and places it in a chair in the room within the view of the patient. The training goes fairly well during the session, and the robot cat is not particularly distracting. After the training, the cat is brought back, and P8 enjoys interacting with it some more.

As P8 enters the room for the second session with the robot cat present, he is wary and careful around it, tentatively holding his hand toward it and quickly pulling back when the robot cat moves. He focuses on the robot cat but often looks at the present adults (a guardian and the dental hygienist) for guidance. When the robot cat meows, P8 responds by imitating the meow. After a few minutes, the robot cat is removed from the dental chair, allowing P8 to sit down, and the dental hygienist starts the session while the guardian holds the robot cat in their lap, sitting on a chair by the side.

In the following sessions, P8 gets decreasingly wary of the robot cat, but at the same time, the training stagnates, he increasingly resists the dental hygienist, and is less collaborative. P8 largely ignores the robot cat, but during the times when he pays attention to it, he increasingly interacts with it as a toy to play with. This play gets increasingly disruptive and starts to become an activity in itself for P8, competing with the training and the treatment. The robot cat thus becomes an excuse for the patient to flee to and focus on instead of the dental training. This is similar to a helpful feature for some patients struggling with the training; however, in their cases, it serves the purpose of providing respite in a stressful time, whereas it was more a casual excuse for P8. It is unclear if the robot cat is helping the patient with being more comfortable in the situation or if it is only disruptive to the point of preventing training and treatment. This is also a case where it might be relevant to remove the robot cat.

In both these cases, it appears likely that the existing tools and routines worked well enough to handle the situations, allowing the training to progress. The introduction of the robot cat disrupted the situation in a way that appeared to have a net negative effect on the training and treatment. For both these patients, but particularly for P8, it is possible that they were not sure exactly why they were in the dental care situation and what was expected of them (despite the different tools and techniques used to try to inform and explain), making it difficult for these patients to understand why they were encouraged to interact with the robot cat yet not allowed to choose the activity over “sitting still and having a person doing things with their teeth.”

### 4.3 Communication modalities

**FIGURE 9:**
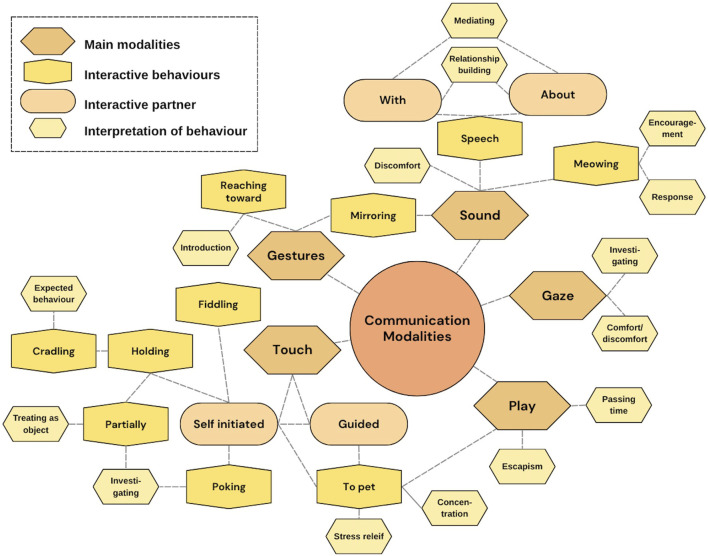
Communication modalities observed in the interaction between patients and the companion robot.

The main communication modalities that were used by the patients were *touch*, *sound*, *gaze*, and *gestures* (see Figure 9). For *touch*, the communication between both the patient and the companion robot was *guided* by either the dental hygienist or the guardian by bringing the patients’ hand toward the robot and *self-initiated*. The interactions were *to pet* the robot for play, to increase concentration, or for stress relief, *poking* at the robot for investigation and *fiddling* with the robot while sitting in the chair. The patients were also *holding* the robot, either *partially* for investigation or by treating it as an object rather than an agent. Some patients were also *cradling* the robot, which could be seen as an expected behavior when holding a cat.

For *sound*, the patients were sometimes *meowing* at the robot cat, either as a response for a previous meow by the robot or as an encouragement toward the robot to do something (e.g., meow or turn on its back). This was an indication that the companion robot was seen as something more than a stuffed animal as the patients expected the robot to respond to them. Even if several of the children were nonverbal, some of the patients either *spoke* with the robot or about it, which sometimes also was guided or encouraged by the adults in the room. This was an interactive process for mediating or as relationship-building with the robot. Some of the sounds made were also a sign of discomfort, where not all patients appreciated the robot making sounds and got distracted by it, especially during the first session. *Gestures* made were for mirroring what the robot was doing, which was the same as for some of the sounds. Another gesture was reaching toward the robot as a kind of introduction. *Gaze* toward the robot was mainly performed by patients to first investigate the robot and second as an expression of seeking comfort from the robot or showing discomfort with the situation.

Finally, we have also included *play* as one of the communication modalities, as, for some of the patients, it was a central way of interacting with the robot cat. One thing that should be noted here was that play was not specifically encouraged by the dental hygienist (e.g., the familiarization method of free play (Wallbridge et al., 2024)) but was also not discouraged when the patients started playing with the robot cat. Some of the patients were using play mainly when the dental hygienist and the guardian were discussing the dental care after the training or treatment, seemingly for passing time. Others, as described in Section 4.2.3, were disrupting the training or treatment session by playing with the robot as a kind of escapism from the tasks that were at hand.

## 5 Discussion

In this exploratory study, we show that a robot cat can in some cases support children with ASD in SDC, but the findings highlight significant individual variations in how the robot was experienced, shaped by the context, timing, and emotional state. The robot’s role was not universally positive or passive; its effectiveness depended on how it was integrated into personalized care strategies by the dental hygienist, guardians, and the patients themselves. Over the year, all patients interacted with the robot cat. Most of the patients interacted with it already at the first session. Previous research has shown that it is unusual for children with ASD to immediately interact with new stimuli, but another study with robot cats found similar patterns (Kwon et al., 2015). After the final session, all guardians expressed a wish to continue using the robot cat, where several also saw a need to include it in other domains (such as at the hairdresser and health centers). One guardian even considered to purchase the robot for the home environment. The positive view by the guardians on the intervention partly speaks in favor of the robot but also for the need for help they see for their children. In this paper, we highlight both the treatment perspective and the different stakeholders’ perspectives, and it is important to remember that the deployment of a companion robot is not a solution for dental treatment itself but a potential aid for some children with ASD during all stages from being in the dental situation to undergo training and treatment as a tool in the toolbox. In the following section, we will discuss how this deployment affected the dental practice.

### 5.1 Affecting the practice

New technology has the potential to be disruptive (for better or for worse). It is, therefore, important to assess what impact it may have before deploying it. However, such assessment is not simply a task to perform once before deployment but needs to be carried out continuously even beyond deployment. Situations, practices, and circumstances can change dynamically, and the technology and the use of technology will need to be adjusted to handle that. This is particularly important when new patients are involved, and identifying which specific practices and tools best fit each patient is still at an early state. Instead of assuming some hypothetical generic benefit of the technology, it is more useful to take the patients’ perspective into account as far as possible and consider what is most relevant and beneficial given their specific situation, desires, and needs. For those reasons, we propose that reflective methods and salutogenic perspectives are particularly helpful when facilitating the appropriate use of the robot in the care situation. When analyzing the data, there were several aspects related to the impact on the practice that stood out. They relate both to the impact on the dental hygienist’s workplace and on the SDC practices.

#### 5.1.1 Disrupting the workplace

For the introduced robot to be meaningful, it needs to be assimilated into the existing practice and be actively facilitated by the care providers. The introduction of a new tool like this is a process in which the dental hygienist tries to introduce it in some way and, based on the response of the patient, adjusts the practice dynamically. To do so, the dental hygienist needs to have an idea or expectation of how the robot might be perceived by the patient and what purpose the robot might serve in the care practice. For instance, the work practice was affected already before the first session, as the information provided to the patient needed to be adjusted to inform them about the robot cat. The initial presentation of the robot will, thus, happen before the first meeting with it, and the dental hygienist will have to identify how to frame it most appropriately in text and images by then. This can be seen as a preparation for facilitating static exploration of the robot cat for the patients (Wallbridge et al., 2024). In our observations of the actual initial sessions, the dental hygienist typically introduced the robot cat by having it lying on the dental chair, wrapped in a blanket, when the patients entered the room. This way, it did not appear suddenly for the patients, and it conveyed a sense of relaxation and calm. The robot cat was left for the patient to discover by themselves, but the dental hygienist was also present and encouraging, facilitating the patients’ discovery of the robot cat.

In the (especially initial) interactions between the patient and the robot cat, the adults in the room spent time to allow the patient to get accustomed to the robot cat. This was sometimes done simply by being close to the robot cat and talking about it. It was also common to be even more active, either by showing how to potentially interact with it (petting it), presenting it to the patient, or holding the patients hand and guiding the hand to the robot cat. Some of these kinds of activities can be considered as a kind of capability demonstration, showing and encouraging potential ways of interaction (Wallbridge et al., 2024).

It was common that each session had some kind of (implicit) negotiation between the patient and the other humans related to what the robot cat could provide (and how) each session. Part of this negotiation constituted a kind of capability demonstration, but throughout the sessions, the dental hygienist would typically propose to remove, add, or change the interaction with the robot cat. This way, the robot cat became increasingly similar to a tool for the dental hygienist as their familiarity with it increased. Although the robot cat’s function in this context is largely to provide comfort for the patient, there were instances where the dental hygienist decided to remove the cat despite the patient enjoying it (which, for instance, occurred with P7). The reason for that was typically because it disrupted some of the important activities too much, without providing the relevant kind of support.

As mentioned in the case descriptions, there were different ways the robot cat was used. It was sometimes useful during training or treatment, for example, as comfort, distraction, or simply as a way for the patient to keep their hands busy. Some patients showed little or no interest in the robot cat during the sessions but engaged with it afterward. In these cases, the patients might have seen the interaction with the robot cat more like a reward afterward and might have found it distracting in an unhelpful way during the session. Some patients also liked to interact with the robot cat while waiting for their guardian and the dental hygienist to speak^4^.

#### 5.1.2 Disrupting relations

The introduction of the robot cat, designed to convey a social presence, means that the situation now contains an additional agent for the others to relate to. Introducing a robot in an existing network of relations will not only introduce new relations (between the robot and the respective humans) but can also affect the relations between the humans (Gillet et al., 2024). One of the fundamental ideas behind introducing the robot was the hope of an interaction-shaping effect. The patient–robot interaction was partly intended as a means to the end of improved patient–dental hygienist interaction, and this effect was observed in all cases, albeit more superficially in some of the cases, which is an effect that other studies have indicated as well (Ali et al., 2020; van Otterdijk et al., 2020). In some of the more superficial cases, the robot cat might have provided a different topic to talk about while there was no shortage of such topics, and the nature of the conversations were not meaningfully altered. However, in other cases, the introduction of the robot cat as a conversational topic did have more impact. For instance, P7 already spoke a lot in the baseline session, but when the robot cat was introduced, the conversations shifted in nature in a way that became distracting for the dental hygienist. In contrast, P3 was able to involve the robot cat in his interactions with the dental hygienist, facilitating smoother and more successful communication. Other studies have also found that children with ASD were more engaged, responsive, and absorbed more information overall when a robot was present (Shahriar et al., 2021).

The robot cat also constituted a tool for the guardian to participate in a different way in some cases. The guardian could in some cases have a more active role of the care situation by physically bringing and removing the robot cat when necessary and could also assist by engaging in conversations about the robot cat. It is also possible that the robot cat might have provided some comfort or support for the guardians themselves, which was confirmed in the post-session interviews with the guardians (Thunberg et al., 2025^3^).

The effects of the robot cat also expand beyond the sessions themselves, both in time and space. The robot cat was part of the information material used by the guardians when preparing the patients at home for upcoming sessions. Some patients also had their pictures taken (or video snippets recorded) with the robot cat, which was sometimes used as part of the preparation for the next session. However, importantly, this material was often also used to show friends and family the robot cat, and there were occasions where the patients’ siblings or friends came along for a session to be able to see it for themselves. This way, the robot cat helped to change the entire context of the dental care from something potentially negatively loaded to something positive, where the patient could feel pride and feel that they have something to show that other people found genuinely interesting. It is, therefore, important to acknowledge the impact of the robot cat at a much larger scale than the sessions themselves, as the effects go further beyond that. This is important even if the interest is strictly on the dental care, as some of the effects beyond the sessions might feed back to the dental care due to, for instance, changing attitudes, as also seen in previous clinical research using robots for children with ASD (Pinto-Bernal et al., 2022).

#### 5.1.3 Caring for the robot

The dental hygienist’s profession is about caring for children and their dental needs. However, care is a complex and situated process that takes place in a context with many nuances that can change quickly (Tronto, 1998). The practice often involves a kind of tinkering, where professional experience and empathy guide the many decisions that are made. It is, however, not only the patient that is the target of the care. It is important for the caregiver to care for their tools and the environment in which the treatment takes place to facilitate good care of the patient. For the robot cat to work, it needs to have its batteries charged, and it needs to be monitored over time to make sure that it gets the maintenance it needs. The dental hygienist made sure that the robot cat had a blanket to lie on and be wrapped in. This was on the one hand a part of the story-telling of conveying relaxation and comfort to the patient but on the other hand also was a way to provide care to the robot cat. As an artificial agent, the robot cat becomes a particular entity in the care situation, both due to its technical nature and to its social purpose (DeFalco, 2020). The patients, guardians, and the dental hygienist ascribed agency to the robot cat, which makes it one of the caring agents in the dental situation.

There were also some care steps that had to be taken for the robot itself. The dental hygienist’s robot wrangling was crucial in the process of making the robot work (Suchman, 2006) by showing the patient how to hold and pet the robot cat and guiding the patients to seek comfort in the robot during stressful moments. She further had to care for the practical aspects of deploying the robot, such as changing batteries and wrapping it in a blanket to alleviate the initial reactions to the deployment and ease interaction.

### 5.2 Limitations

We acknowledge that some of the effects observed in the dental sessions could have other explanations than due to the robot intervention, such as the *age*, *new medication*, *diagnoses*, and *additional tools*. Given the young age of the patients in our study, it is worth highlighting that the study has taken place over a large part of their lives (more than 15% at the point of the final recording for some patients) and that this age is particularly formative with a lot of development occurring in general for them. It can, for that reason, be difficult to identify what progress in participating in dental care is due to the robot cat and what is due to general development, especially for the youngest of the patients. In addition, as can be seen in Table 1, most of the patients are regularly using medication, some of which was introduced or adjusted throughout the year of our observations, which might have been observed as an effect of improvements from one session to another. It should also be noted that several of the patients have multiple diagnoses, some of which can only be diagnosed properly through continuous observation of cognitive development that comes with older age. Another point to highlight is the additional tools or strategies that the patients and their families sometimes relied on, beyond what was offered by the dental care facility. However, not many of the patients used tools other than “tell–show–do,” visual support, counting down, singing, applauses, and a small toy reward. One patient used a puzzle, one a fidget spinner, and one used earphones, but only at one session each.

An additional limitation was that all the participants were boys. We recognize that there is a widespread exclusion of girls in autism research (D’Mello et al., 2022) even though the ratio for ASD between boys and girls is equal (Burrows et al., 2022). This inequality in diagnosis might cause the vast over-representation of boys among the children accessing this kind of care, which in turn constituted our pool of potential participants.

We have also intentionally stayed away from quantitative measurements and analysis in this study, both because our focus was on the variation rather than the norm and due to the care professionals’ worry that quantitative metrics would warp the focus of the care situation to something where the needs of the patients become secondary. For instance, the session times could have been affected by the introduction of the robot cat; however, introducing that variable insinuates that the session length has a value in itself, whereas the actual priority is on good care.

## 6 Conclusion

In this exploratory study, we examined whether a robot cat could support children aged 5–10 years with ASD during dental visits over the course of a year. Although some patients benefited from the robot as a means of easing anxiety and enhancing cooperation and a feeling of safety, others found it to be a neutral or even disruptive element in their training or treatment, where too much focus was directed toward the robot that it became a distraction. In this paper, we emphasize the complexity of identifying behaviors based on the context, timing, and emotional state, reflecting the dynamic nature of the patient’s ongoing development. This is particularly relevant for children with one or multiple neuropsychiatric disabilities, where tailored approaches are crucial for effective support. One robot platform does not fit all, and the results show how care is mediated through the robot by all parties, that is, the dental hygienist, the guardians, and the patients themselves, to effectively make the complex dental training situation work for all. It is also clear that the robot is not a passive quick-fix; it has to be actively integrated into the practice based on the particularities of the specific context and the particular people involved. Future research should continue to explore how such technologies can be optimized for individualised use, ensuring that they effectively complement existing behavioral and psychosocial interventions.

## Data Availability

The datasets presented in this article are not readily available because of our ethical protocol. Further enquiries can be directed to the corresponding author.

## Footnotes

1
https://rkrattsbaser.gov.se/sfst?bet=1985:125

2
https://rkrattsbaser.gov.se/sfst?bet=2001:453

3 Thunberg, S., Jönsson, A., and Sundell, A. L. (2025). The role of a robot cat in supporting children with autism spectrum disorder during dental visits: an exploratory study. Int. J. Paedatric Dent. Submitt.

4 It is common that a conversation is had between the adults after the training and treatment. In these conversations, the dental hygienists can highlight aspects they have discovered, provide support and answer questions regarding domestic dental care, and schedule the next session.
